# Deciphering the Subtype Differentiation History of SARS-CoV-2 Based on a New Breadth-First Searching Optimized Alignment Method Over a Global Data Set of 24,768 Sequences

**DOI:** 10.3389/fgene.2020.591833

**Published:** 2021-01-11

**Authors:** Qianyu Lin, Yunchuanxiang Huang, Ziyi Jiang, Feng Wu, Lan Ma

**Affiliations:** ^1^Tsinghua-Berkeley Shenzhen Institute, Tsinghua University, Shenzhen, China; ^2^Tsinghua Shenzhen International Graduate School, Tsinghua University, Shenzhen, China; ^3^Shenzhen Bay Laboratory, Shenzhen, China

**Keywords:** SARS-CoV-2, multiple sequence alignment, phylogenetic tree, t-SNE, haplotype network analysis

## Abstract

SARS-CoV-2 has caused a worldwide pandemic. Existing research on coronavirus mutations is based on small data sets, and multiple sequence alignment using a global-scale data set has yet to be conducted. Statistical analysis of integral mutations and global spread are necessary and could help improve primer design for nucleic acid diagnosis and vaccine development. Here, we optimized multiple sequence alignment using a conserved sequence search algorithm to align 24,768 sequences from the GISAID data set. A phylogenetic tree was constructed using the maximum likelihood (ML) method. Coronavirus subtypes were analyzed via t-SNE clustering. We performed haplotype network analysis and t-SNE clustering to analyze the coronavirus origin and spread. Overall, we identified 33 sense, 17 nonsense, 79 amino acid loss, and 4 amino acid insertion mutations in full-length open reading frames. Phylogenetic trees were successfully constructed and samples clustered into subtypes. The COVID-19 pandemic differed among countries and continents. Samples from the United States and western Europe were more diverse, and those from China and Asia mainly contained specific subtypes. Clades G/GH/GR are more likely to be the origin clades of SARS-CoV-2 compared with clades S/L/V. Conserved sequence searches can be used to segment long sequences, making large-scale multisequence alignment possible, facilitating more comprehensive gene mutation analysis. Mutation analysis of the SARS-CoV-2 can inform primer design for nucleic acid diagnosis to improve virus detection efficiency. In addition, research into the characteristics of viral spread and relationships among geographic regions can help formulate health policies and reduce the increase of imported cases.

## Introduction

Severe acute respiratory syndrome coronavirus 2 (SARS-CoV-2) is a novel coronavirus, which is the etiologic agent of the disease, coronavirus disease 2019 (COVID-19) (Li et al., 2020). SARS-CoV-2 emerged in late 2019 in Hubei Province, China (Chen et al., 2020; Zhou et al., 2020), and spread worldwide with incredible rapidity, resulting in a global pandemic. As of October 13, 2020, more than 38 million people have been infected worldwide with approximately 1,090,000 deaths. The number of newly diagnosed cases has increased dramatically with tens of thousands confirmed daily. In Europe, the case fatality rate exceeded 7%, and those in France and Belgium have reached unprecedented levels at 24.5 and 33.4%, respectively (World Health Organization, 2020; Worldometer, 2020).

Analysis of virus mutation sites is necessary for applications, including vaccine development (Ma et al., 2020) and primer design for virus nucleic acid detecting. It is reported that conserved sequence-based mRNA vaccines (Freyn et al., 2020) and peptide vaccines (Herrera-Rodriguez et al., 2018) have successfully made the vaccinated generate immunity to multistrains of the same virus. The conserved sequences have great potential in long-acting vaccine design. Multiple sequence alignment (MSA) methods are invariably used for automated identification of mutation sites and widely used in SARS-CoV-2 sequence analysis (Lai et al., 2020; Wu et al., 2020) in the early stage of the pandemic. With the fast increase of SARS-CoV-2 sequencing data, it is significant to improve the efficiency of the current MSA algorithms to fit the large-scale data set. We developed a new method for conserved sequence searching. Large data sets containing long sequences, such as the SARS-CoV-2 data set, can be optimized by pruning conserved sequences to fit current MSA algorithms. MSA methods invariably detect conserved sequences, and, using our approach, conserved sequence identification is independent of MSA.

Using data from the GISAID database (Elbe and Buckland-Merrett, 2017), we analyzed COVID-19 strains from around the world on an unprecedented scale. All the mutations in SARS-CoV-2, including 33 sense, 17 nonsense, and amino acid loss/insertion mutations, were identified using MSA. Further, based on the results of MSA, we constructed phylogenetic trees and used the t-SNE method to cluster SARS-CoV-2 subtypes. Our findings demonstrate the characteristics of viral spread and uncover relationships among countries and continents.

## Materials and Methods

### Data Source and Data Selection

The SARS-COV-2 sequences used in this study were all collected from the GISAID (Elbe and Buckland-Merrett, 2017) database and were download on May 14, 2020.

To identify mutations in full-length sequences and determine global spread relationships, the download parameters were set as, “complete(>29,000 bp)” and a total of 24,768 sequences were retrieved. According to codon table and DNA translation rules, sequences were compared with annotations of NC_045512.2 from NCBI (Benson et al., 2018) and high-quality open reading frame (ORF) regions with no degenerate bases (including N) translated into amino acid sequences for each record. The number of sequences for each ORF are shown in Supplementary Table 1. Further, 9,308 sequences with 12 full-length, high-quality ORF regions and a clear collection date were available for use in building phylogenetic trees and t-distributed stochastic neighbor embedding (t-SNE). The coding language used was Matlab (R2020a for windows).

### Conserved Sequence Searching

A new strategy to evaluate conserved sequences was developed based on the breadth-first search algorithm. The search queue “q” was initiated using 20 one-length protein segments (single amino acid). Considering *q*_*i*_ as the *i*th string in the queue and *d*_*j*_ as the *j*th sample sequence of the data set, *p*_*i*_, Eq. 1 was used to evaluate the probability of conservation:

$$p_{i}=\frac{\sum_{j=1}^{n}f\,\left(q_{i},d_{j}\right)}{n},f\,\left(q_{i},d_{j}\right)=\left\{ \begin{array}{c} 1\,\,i\,f\,q_{i}\,a\,n\,d\,d_{j}\,m\,a\,t\,c\,h\\ 0\,\,o\,t\,h\,e\,r\,w\,i\,s\,e\; \end{array}\right.\;\left(1\right)$$

**FIGURE 1:**
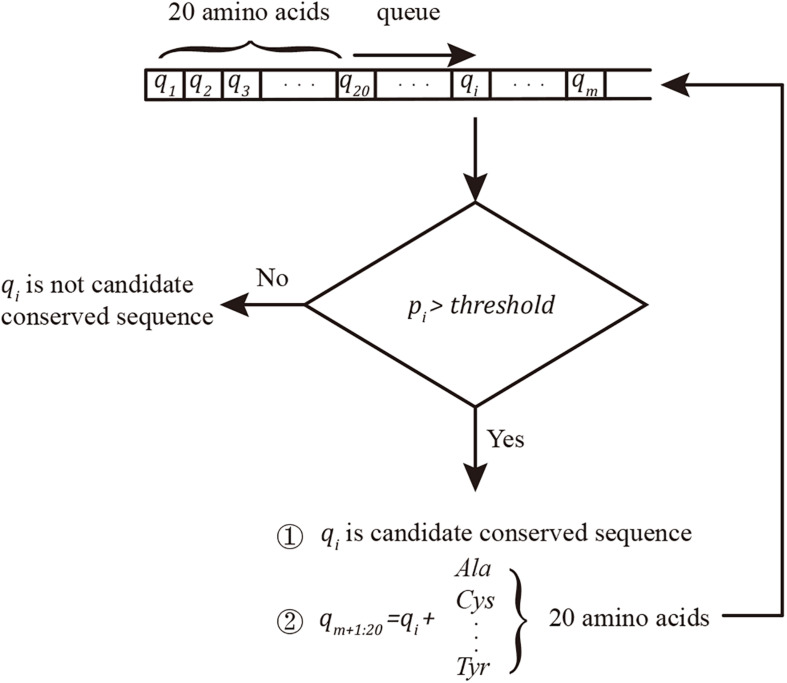
Conserved sequence searching algorithm. The queue was initiated by 20 amino acids. *q*_*i*_ was considered a conserved sequence when *p*_*i*_ ≥ threshold; in this project, the threshold was set as 100%. Twenty new conserved sequence candidates were built by adding new amino acids to the end of the confirmed candidate conserved sequences and added to the end of the searching queue *q*.

The conserved sequence search algorithm (pseudo code in Supplementary Data 1) is shown in Figure 1.

The match function, “strcmp,” was applied in Matlab. In another coding language, the KMP (Knuth et al., 1977) algorithm can improve the string matching speed. In this approach, the conserved sequences in queue *q* with length >5 can avoid ectopic repeats.

### Multiple Sequence Alignment (MSA) and Mutant Site Analysis

Multiple sequence alignment was conducted for each ORF data set. Identification of conserved sequences can efficiently separate long sequences into short segments, which can greatly reduce the alignment time cost so that it reaches a tolerable level. The MSA function used was “multialign” in Matlab. Statistical analysis of mutant sites was conducted directly using aligned sequences.

### Phylogenetic Tree Construction and Haplotype Network Analysis

We constructed the maximum likelihood (ML) tree using Raxml-ng (Kozlov et al., 2019) (v.0.8.0 BETA) software.

Sample sequences (*n* = 9,308) with full-length translated ORF and clear collection date were selected; however, these were still too long [length > 7,000 nucleotides (nt)] for use in MSA; in general, samples with long sequences and large data sets lead to excessively high time costs for phylogenetic tree construction. Therefore, sequence pruning was necessary. According to Shannon’s information theory (Shannon, 1948), the information entropy of any segment in sequence can be calculated by Eq. 2:

$$H_{i}=p_{i}\times \log\left(p_{i}\right)\,\left(2\right)$$

Clearly, if conserved sequence *q*_*i*_ with *p*_*i*_ → 100% has information entropy *H*_*i*_ → 0, it can be easily proven that deleting *q*_*i*_ from all sequences will not substantially influence the results of phylogenetic analysis.

Considering the limitations of phylogeny performance time cost and visualization, we pruned sequences by deleting highly conserved bases with *p*_*i*_ > 99.9%. Pruned sequences with exactly the same sequence were reduced to 1 as a representative, resulting in a final selected 1,291 samples.

We performed 50 tree searches using 25 random and 25 parsimony-based starting trees on each DNA data substitution matrix in Raxml-ng, and we got a ML and lowest AIC/AICs/BIC score with GTR + GA model. One thousand bootstrap replicates with seed 2020 were conducted and the transfer bootstrap expectation (tbe) metric was calculated to map onto the best-scoring ML tree to generate proportional support values.

Phylogenetic trees were visualized using iTOL (Letunic and Bork, 2019). Larger clade naming rules refer from GISAID (S: C8782T, T28144C; L: C241, C3037, A23403, C8782, G11083, G25563, G26144, T28144, G28882; V: G11083T, G26144T; G: C241T, C3037T, A23403G; GH: C241T, C3037T, A23403G, G25563T; GR: C241T, C3037T, A23403G, and G28882A). It is worth noting that the marker variant C241T for clade identification is not included in ORF region. We count the C241T base frequency in each haplotype and give the C241T base information lost samples an inferred subtype if 100% frequency base exist; otherwise, the samples will be labeled as “Other.”

The haplotype map with median-joining network (Bandelt et al., 1999) was created by PopART (version 1.7) (Leigh and Bryant, 2015), and 9,308 full ORF region sequenced samples are identified into 300 haplotypes by 77 variant sites (*p*_*i*_ < 99.5%). For better visualization and clearer topology of the haplotype network, we deleted the haplotypes with a single case, and in total, 153 haplotypes are used in haplotype network construction.

### t-Distributed Stochastic Neighbor Embedding (t-SNE)

Samples with 12 high-quality, full-length ORF regions, and a clear collection date (*n* = 9,308) were subjected to t-SNE unsupervised clustering. The t-SNE function used was “tsne” in Matlab. Results were visualized using the “gscatter” function in Matlab. The distance function used was the PAM250 matrix (for amino acid sequence) and BLOSUM45 matrix (for nucleotide sequence). For each aligned amino acid sequence, each amino acid was considered as a dimension of the sample. The distance between sample *S*_*i*_ and *S*_*j*_ was calculated using Eqs 3 and 4:

$${\mathrm{Distance}}_{i,j}=\frac{\sum_{k=1}^{\mathrm{length}\,\left(S\right)}\mathrm{PAM}\,250\,\left(S_{i}\,\left(k\right),S_{j}\,\left(k\right)\right)\,\wedge \,2}{\mathrm{length}\,\left(S\right)}\;\left(3\right)$$

$${\mathrm{Distance}}_{i,j}=\frac{\sum_{k=1}^{\mathrm{length}\,\left(S\right)}\mathrm{BLOSUM}\,45\,\left(S_{i}\,\left(k\right),S_{j}\,\left(k\right)\right)\,\wedge \,2}{\mathrm{length}\,\left(S\right)}\;\left(4\right)$$

In Eq. 3, *S*_*i*_(*k*) is the *k*th amino acid of the amino acid sequence *S*_*i*_. In Eq. 4, *S*_*i*_(*k*) is the *k*th base of the nucleotide sequence *S*_*i*_. To reduce overlap, the final coordinate of each sample was adjusted to a short radius from the origin position, which did not influence cluster information.

## Results

### Mutations of SARS-CoV-2 ORF Regions

SARS-CoV-2 full-length nucleotide sequences (*n* = 24,768) were collected from GISAID up to May 14, 2020, and translated into amino acid sequences. Due to the presence of degenerate bases, the size of the available high-quality amino acid sequence data set for analysis of mutant sites was <24,768. The final sequence set size was as follows: ORF1a (*n* = 16,863), ORF1b (*n* = 14,252), S (*n* = 16,851), ORF3a (*n* = 23,390), E (*n* = 24,344), M (*n* = 23,513), ORF6 (*n* = 24,199), ORF7a (*n* = 21,690), ORF7b (*n* = 21,953), ORF8 (*n* = 24,288), *N* (*n* = 23,176), and ORF10 (*n* = 24,043).

**FIGURE 2:**
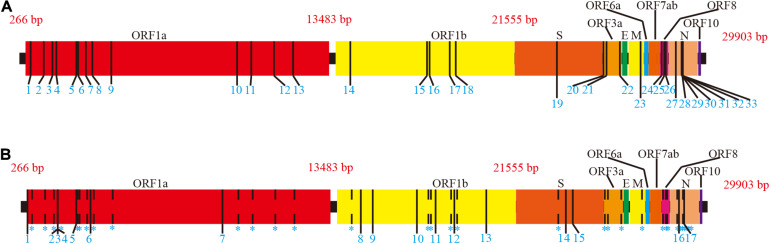
Mutations of full length on SARS-CoV-2 ORF region. The NC_045512.2 SARS-CoV-2 sequence from NCBI was used as the reference. Adjacent ORF areas are distinguished by different colors. **(A)** Sense mutation sites. Mutation sites are marked with a thin black vertical line. Details of sense mutant sites are presented in Supplementary Table 2. **(B)** Nonsense mutation sites. Sense mutation sites are indicated by dashed lines for comparison. Nonsense mutation sites are marked as thin, black vertical lines. Details of nonsense mutant sites are presented in Supplementary Table 3.

According to the MSA of each ORF region (available in Supplementary Data 2), 50 mutation sites with frequencies >1% were detected, including 33 sense and 17 nonsense mutation sites (Supplementary Tables 2, 3 and Figure 2).

Mutations were present in the ORF1ab, S, ORF3a, M, ORF8, and *N* regions with more than half of mutations in the ORF1ab region. Given the differences in length of ORFs, ORFs with a higher proportion of mutations (number of mutation sites/ORF length) were ORF8 (2.48%), ORF3a (1.09%), and N (1.67%). ORFE, ORF6, ORF7ab, and ORF10 were completely conserved across the entire length of the ORF region. Notably, the S region, which is the SARS-CoV-2 antigen recognition protein, contained only one sense mutation site D614G encoded by A23403G; hence, current data indicate that the S region is highly conserved. Although the D614G spike protein variant has proved it is more infectious than D614 strains (Korber et al., 2020; Yurkovetskiy et al., 2020), it is equally sensitive to neutralization by monoclonal antibodies targeting the receptor-binding domain (Yurkovetskiy et al., 2020).

Amino acid loss and insertion mutations are listed in Supplementary Tables 4, 5, respectively. The location referred to in these tables is based on the NC_045512.2 nucleotide sequence as a prototype. As shown, the majority of amino acid loss and insertion mutations only occurred in a single sample although loss mutation No. 7 and insertion mutation No. 1 had higher frequencies than other mutations of this type.

### Phylogenetic Trees and Haplotype Analysis

**FIGURE 3:**
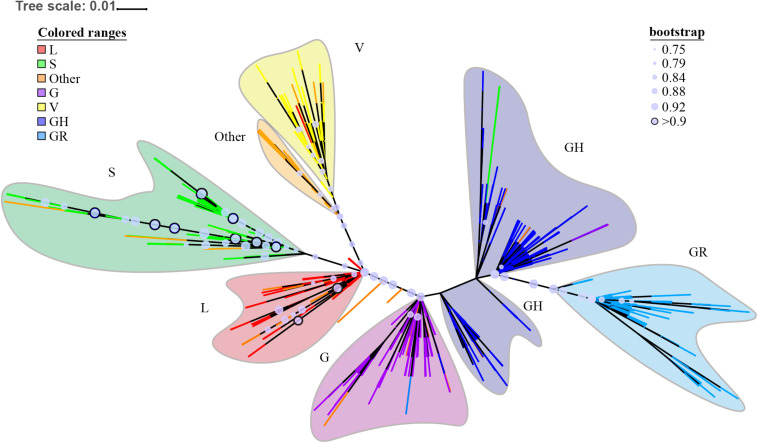
ORF region based maximum likelihood (ML) phylogenetic tree. The ML tree is displayed in unrooted mode; the deepest branches are colored to represent different subtypes. Major lineages are colored and named. Bootstrap support values are indicated by circles on nodes for support of 0.75 and above. The circles with bootstrap support values over 0.9 are highlighted by a black border. Label information is present in Supplementary Figure 1, and in Newick format in Supplementary Data 3. Configuration files for iTOL visualization are also in Supplementary Data 3.

The ML phylogenetic tree is shown in Figure 3. The complete phylogenetic tree in normal format with bootstrap support value and leaf labels is shown in Supplementary Figure 1. The large clade branches have high tbe-supported values (>0.75). Deeper branches’ tbe-supported values are sometimes lower. We have similar main group results as the neighbor-joining (NJ) tree in GISAID; what is different from GISAID’s NJ tree is that our results show clades G/GH/GR are closer to the root than clades S/L/V.

**FIGURE 4:**
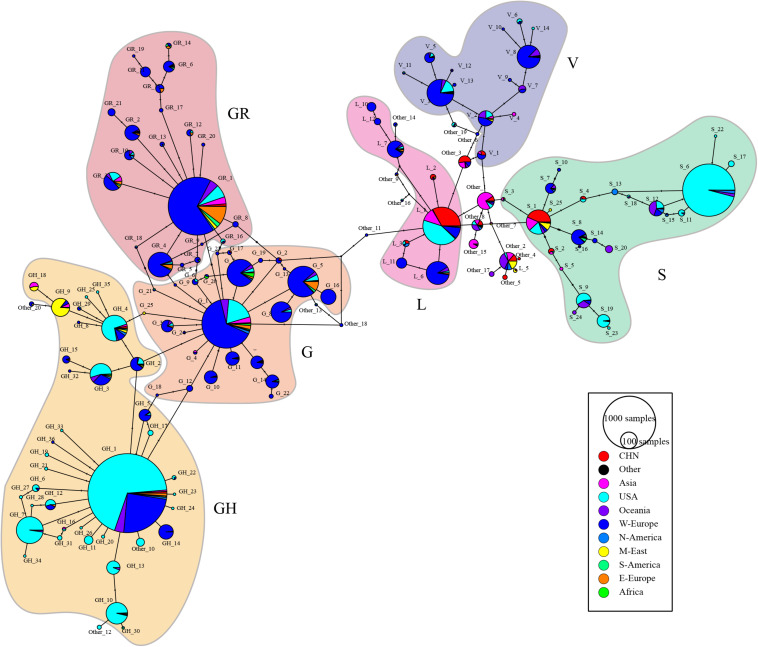
ORF region based median-joining haplotype network map. The black circles are the median vectors; circle size is proportional to the numerosity of the haplotypes. Each haplotype appears as a pie chart and is colored according to the geography distribution of the haplotype. Mutations are shown as hatch markers. Haplotypes are labeled in the format of “subtype_order,” and Supplementary Table 6 list the prototype sequence of each haplotype.

Figure 4 depicts the median-joining network haplotype result. Haplotypes L1, S6, V3, G1, GR1, and GH1 are the biggest haplotypes of their belonging clades. Clade S and clade G play important roles in coronavirus strain differentiation. Compared with big haplotypes in clades L/S/V, big haplotypes in clades G/GH/GR have more connections to other haplotypes, consistent with the fact that wider spreading will inevitably provide more mutant opportunities and, thus, lead to more sub-haplotypes and haplotype connections.

### t-SNE Unsupervised Clustering Reveals International Spread Relationships

The t-SNE method is widely used in single-cell RNA sequencing investigations to cluster different cell types. In this study, t-SNE was used to cluster coronavirus gene subtypes according to amino acid sequence.

**FIGURE 5:**
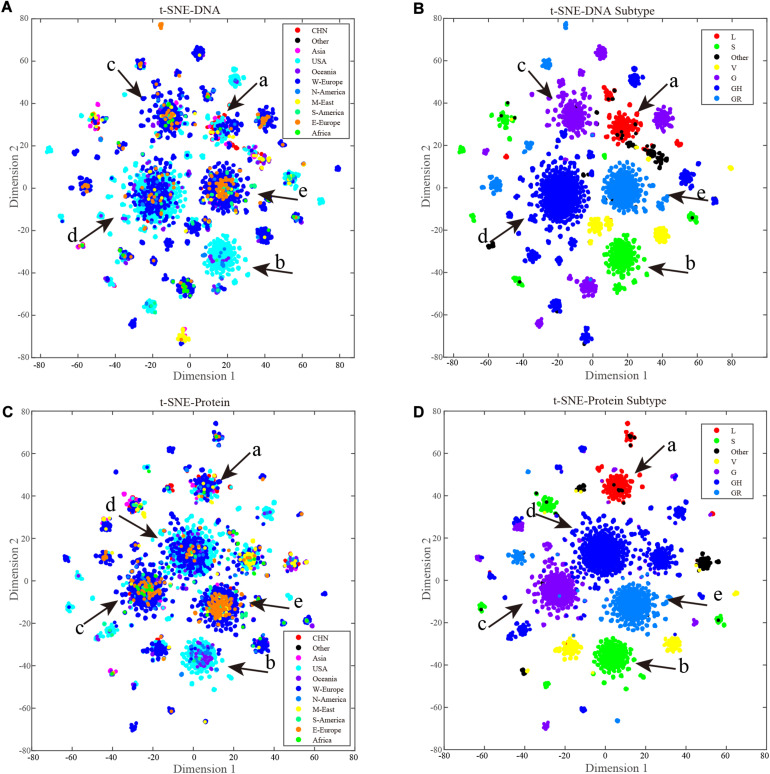
ORF region based t-SNE clustering. Each point is a sample, and point colors represent the sample source. A key is provided in the upper right corner of the figure. The main clusters are marked from a to f with red arrows. **(A)** A total of 9,308 full length ORF region nucleotide sequences t-SNE results, points are colored by geography labels. **(B)** A total of 9,308 full length ORF region nucleotide sequences t-SNE results, points are colored by clade labels. **(C)** A total of 9,308 full length ORF region amino acid sequences t-SNE results, points are colored by geography labels. **(D)** A total of 9,308 full length ORF region amino acid sequences t-SNE results, points are colored by clade labels.

Each cluster can be considered as a subtype, and labeling the samples according to current clade naming rules, t-SNE clustering does have good performance in sequence subtype identification, at both the nucleotide (Figure 5B) and amino-acid levels (Figure 5D). Samples in the same subtype are more closely related in terms of spread characteristics. Labeling the samples by geographical information (Figures 5A,C), as the figures show, cases from China were mainly concentrated in cluster a, and cases from the United States were present in all main clusters. Most of the smaller clusters as well as most cases in cluster b were from the United States. Clustering of western European cases coincided with those from the United States, indicating that their spread relationships were closer than those of others. Compared with other countries/continents, cases in the United States and western Europe appeared to include more clusters, indicating more sources of spread or a longer history of mutation accumulation.

**FIGURE 6:**
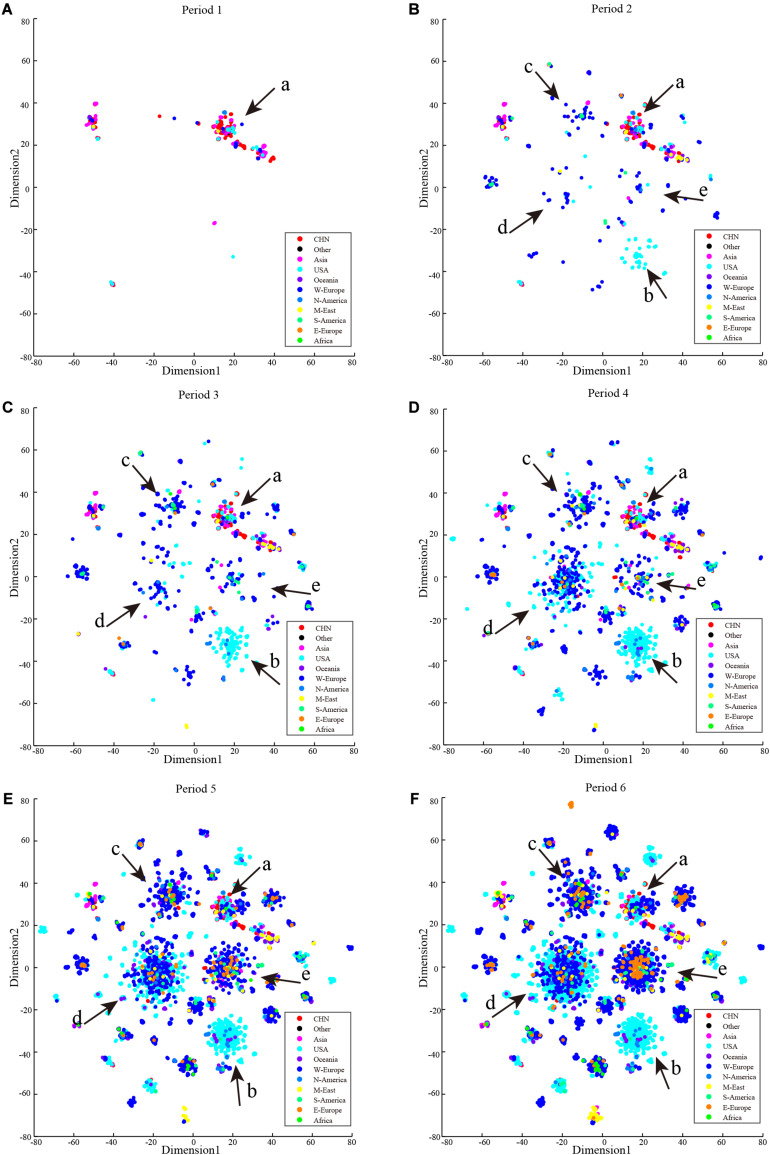
Period t-SNE result. t-SNE results according to collection date order. **(A)** Collection date from December 24, 2019 to February 2, 2020. **(B)** Collection date from December 24, 2019 to March 4, 2020. **(C)** Collection date from December 24, 2019 to October 3, 2020. **(D)** Collection date from December 24, 2019 to March 16, 2020. **(E)** Collection date from December 24, 2019 to March 25, 2020. **(F)** Collection date from December 24, 2019 to April 5, 2020.

Cluster development and the process of COVID-19 spread in recent months are shown in Figure 6. Cluster b contained only cases from the United States in the early stage of the pandemic, and it also contained other North American and Oceania cases in subsequent months. Cases in cluster a showed a limited increase, and those in clusters b, c, d, and e have grown rapidly. Compared with the early stages of the pandemic, the number of clusters has not increased substantially with the main mutations in SARS-CoV-2 occurring before March 18, 2020.

## Discussion

### Conserved Sequence Searching and MSA Optimization

Traditional conserved sequence analyses rely on MSA tools, such as ClustalW (Larkin et al., 2007), MUSCLE (Edgar, 2004), and T-coffee (Di Tommaso et al., 2011). ClustalW calculates a distance matrix by pairwise alignment, builds a guide tree, and makes progressive alignment based on the guide tree. It is the most widely used tool for MSA, but it is also the slowest. T-coffee generates more accurate results than other methods; however, it is more applicable for small data sets (*n* < 100 advised) and short-length sequence data, and the alignment speed is inadequate. MUSCLE is faster than the first two methods; however, it has a high memory requirement and cannot match long sequences. Compared with these current methods, our method using conserved sequence searching runs more rapidly in large data sets under time complexity described by *O*(*NL_sample_L_longest conserved sequence_*). Taking conserved segments as anchors to separate a long sequence into several short sequences can effectively improve the efficiency of traditional MSA methods, which makes them feasible for long sequences and large-scale data sets. Our method can only be applied for conserved sequence searching; however, its implementation can assist in application of other MSA methods for phylogenetic tree construction although it cannot perform this function directly. Further, the optimization approach is best applied to intraspecific data sets as there may be insufficient conserved sequences for pruning in other analyses; however, for applications such as primer design, our method of conserved sequence searching has unparalleled advantages compared with other MSA approaches.

### Necessity of Long-Term Conserved Regions Analysis

In our results statistics, the sense and nonsense mutations in SARS-CoV-2 have occurred up to April 5, 2020. Some research (Zhou et al., 2020; Yu et al., 2020) discusses the relationship between SARS-CoV-2 and bat SARSr-CoVs although we only focus on intraspecific subtype differentiation and mutations of SARS-CoV-2. Compared with other research on SARS-CoV-2 mutations (Kim et al., 2020; Ugurel et al., 2020), we had the same results in the main mutations, we list more rare mutation sites in order to have a more comprehensive presentation, but we did not statistical analyze the mutations on a non-coding region, such as C241T.

The COVID-19 pandemic is lasting; however, its duration is relatively short compared with other viral epidemics, and this epidemic may become a long-term public health event (Kissler et al., 2020). New mutations occurring in currently conserved sequences, even conserved ORF regions, remain possible and will bring new challenges for nucleic acid-based diagnosis and vaccine development. Nucleotide mutations in the coronavirus may result in failures of detection. Therefore, it is necessary to avoid frequently mutated areas when designing primers for nucleic acid diagnosis, and primers should be updated in real time, according to mutations in the viral nucleic acid. Therefore, MSA is important for updating primers used for nucleic acid-based diagnosis and improving detection rates. Hence, continuous MSA analyses of new sequencing data are necessary. The influence of rare mutations prune to phylogeny and haplotype analysis.

To improve the efficiency of phylogenetic tree construction, bases conserved in more than 99.9% of samples were pruned, and although rare mutations may possibly be technical artifacts rather than biological mutations (De Maio et al., 2020), the resolution of the tree is still influenced. In haplotype analysis, we pruned more rare mutations (*p*_*i*_ < 0.5%) for better visualization, which may lose some subtype connections linked by these rare haplotypes. In addition, ignoring the non-coding region is also another kind of sequence over pruning. Because we did not use the non-coding region in phylogenetic tree construction, we would lose information from some important variants, such as C241T on 5′-UTR even though we used many more haplotypes in the phylogenetic tree and haplotype network construction than other research and provided more details in SARS-CoV-2 subtype differentiation in the early stage of the pandemic.

### t-SNE Clustering in Sequence Analysis

The t-SNE method provides a new perspective for sequence data analysis. The comparison (Figures 5B,D) between t-SNE clustering results and current clade identification results prove the good performance of t-SNE in sequence-based subtype identification.

Our t-SNE results clearly demonstrate the relationships among countries/continents in the pandemic (Figure 6); however, the cases that occurred in the early period of the pandemic do not tell the origins of their belonging subtypes. One subtype strain may have already spread widely in another region but not be detected due to limited testing ability. From this perspective, providing universal viral nucleic acid detection capability remains highly desirable for analysis of SARS-CoV-2 and requires international cooperation and information sharing.

## Conclusion

In this research, we developed a breadth-first search-based conserved sequence searching method for MSA optimizing and applied it on GISAID’s SARS-CoV-2 data set for sequence analyzing. Our phylogenetic tree and haplotype network results show that clade S and clade G play important roles in SARS-CoV-2 subtype differentiation history. In addition, we show the feasibility of t-SNE clustering in sequence data-based subtype classification. Overall, our research provides new ideas for sequence analysis, which can provide benefits for SARS-CoV-2 sequence-based researches.

## Data Availability Statement

The original contributions presented in the study are included in the article/Supplementary Materials, further inquiries can be directed to the corresponding author/s.

## Author Contributions

LM conceived, designed, and supervised this study. QL designed the study, coded all the programs, collected, and analyzed the data. QL, ZJ, and FW drafted and checked the manuscript. YH visualized the data and format all pictures and tables. All authors discussed the results and commented on the manuscript. All authors read and approved the final manuscript.

## Conflict of Interest

The authors declare that the research was conducted in the absence of any commercial or financial relationships that could be construed as a potential conflict of interest.
