# Supplementary material for: Deciphering the Subtype Differentiation History of SARS-CoV-2 Based on a New Breadth-First Searching Optimized Alignment Method Over a Global Data Set of 24,768 Sequences
Source: Front Genet. 2021 Jan 11;11:591833. doi: 10.3389/fgene.2020.591833 (PMC7831388; doi:10.3389/fgene.2020.591833)
Supplement: Supplementary file 4 [file Data_Sheet_4.docx]

q[1...20] = Ala(A) to Tyr(Y) % initialize the queue q

front = 0

rear = 20

while rear>front

front = front+1

calculate p[rear]

if p[rear]>threshold %q[pear] is conserved sequence

q[rear+1...20] = q[rear] + Ala(A)...Tyr(Y)

rear = rear+20

end

end
