# Supplementary material for: Deciphering the Subtype Differentiation History of SARS-CoV-2 Based on a New Breadth-First Searching Optimized Alignment Method Over a Global Data Set of 24,768 Sequences
Source: Front Genet. 2021 Jan 11;11:591833. doi: 10.3389/fgene.2020.591833 (PMC7831388; doi:10.3389/fgene.2020.591833)
Supplement: Supplementary file 5 [file Data_Sheet_5.docx]

**Supplementary Figure 1.** ML tree is displayed in normal mode. Labels are named in “GISAID ID | Geography location | Collection date” format. The deepest branches and their label background are colored to represent different subtypes. Bootstrap support value are indicated by circles on nodes for support of 0.75 and above.
