# Supplementary material for: Deciphering the Subtype Differentiation History of SARS-CoV-2 Based on a New Breadth-First Searching Optimized Alignment Method Over a Global Data Set of 24,768 Sequences
Source: Front Genet. 2021 Jan 11;11:591833. doi: 10.3389/fgene.2020.591833 (PMC7831388; doi:10.3389/fgene.2020.591833)
Supplement: Supplementary file 6 [file Data_Sheet_6.docx]

Supplementary Material

# Supplementary Table 1. Processed amino acid sequence dataset size of each ORF region

| **ORF1a** | **ORF1b** | **S** | **ORF3a** | **E** | **M** |
| --- | --- | --- | --- | --- | --- |
| 16863 | 14252 | 16851 | 23390 | 24344 | 23513 |
| **ORF6** | **ORF7a** | **ORF7b** | **ORF8** | **N** | **ORF10** |
| 24199 | 21690 | 21953 | 24288 | 23176 | 24043 |

# Supplementary Table 2. Sense mutation sites in ORF region

| No | Gene | Location | Base Changes (%) | AA Changes (%) |
| --- | --- | --- | --- | --- |
| 1 | ORF1a | 490 | T:A:C= 98.97%:1.03%:0.01% | Asp:Glu:Gly:Asn = 98.96%:1.03%:0.01%:0.01% |
| 2 | ORF1a | 1059 | C:T:A = 79.42%:20.55%:0.02% | Thr:Ile:Asn = 79.42%:20.55%:0.02% |
| 3 | ORF1a | 1440 | G:A = 98.39%:1.61% | Gly:Asp:Cys = 98.38%:1.61%:0.02% |
| 4 | ORF1a | 1609 | C:-:T = 97.06%:2.9%:0.04% | Asp:- = 97.1%:2.9% |
| 5 | ORF1a | 2480 | A:G = 95.88%:4.12% | Ile:Val = 95.88%:4.12% |
| 6 | ORF1a | 2558 | C:T = 95.67%:4.33% | Pro:Ser:Leu = 95.67%:4.32%:0.01% |
| 7 | ORF1a | 2891 | G:A:T = 98.4%:1.6%:0.01% | Ala:Thr:Ser = 98.4%:1.6%:0.01% |
| 8 | ORF1a | 3177 | C:T:A = 98.91%:1.06%:0.04% | Pro:Leu:Gln:Ser:Ile:His = 98.89%:1.05%:0.02%:0.02%:0.01%:0.01% |
| 9 | ORF1a | 4002 | C:T = 98.97%:1.03% | Thr:Ile = 98.97%:1.03% |
| 10 | ORF1a | 9477 | T:A = 98.85%:1.15% | Phe:Tyr = 98.85%:1.15% |
| 11 | ORF1a | 10097 | G:A = 98.06%:1.94% | Gly:Ser:Asp = 98.05%:1.94%:0.01% |
| 12 | ORF1a | 11083 | G:T = 88.08%:11.91% | Leu:Phe:Val = 88.07%:11.88%:0.04% |
| 13 | ORF1a | 11916 | C:T = 98.55%:1.45% | Ser:Leu = 98.55%:1.45% |
| 14 | ORF1b | 14408 | T:C = 67.31%:32.69% | Leu:Pro:Phe:Ser = 67.29%:32.68%:0.02%:0.01% |
| 15 | ORF1b | 17747 | C:T = 92.18%:7.82% | Pro:Leu:Ser = 92.16%:7.82%:0.02% |
| 16 | ORF1b | 17858 | A:G = 92.07%:7.93% | Tyr:Cys = 92.07%:7.93% |
| 17 | ORF1b | 18736 | T:C = 98.69%:1.31% | Phe:Leu = 98.69%:1.31% |
| 18 | ORF1b | 18998 | C:T = 98.8%:1.2% | Ala:Val = 98.8%:1.2% |
| 19 | S | 23403 | G:A = 67.79%:32.21% | Gly:Asp:Asn = 67.79%:32.19%:0.01% |
| 20 | ORF3a | 25429 | G:T:- = 97.13%:2.86%:0.01% | Val:Leu:- = :97.13%:2.86%:0.01% |
| 21 | ORF3a | 25563 | G:T:A:C = 77.35%:22.63%:0.01%:0.01% | Gln:His = 77.35%:22.65% |
| 22 | ORF3a | 26144 | G:T = 89.64%:10.36% | Gly:Val:Cys = 89.63%:10.36%:0.01% |
| 23 | M | 27046 | C:T = 98%:2% | Thr:Met = 98%:2% |
| 24 | ORF8 | 27964 | C:T = 98.16%:1.84% | Ser:Leu = 98.16%:1.84% |
| 25 | ORF8 | 28077 | G:C:T = 98.77%:1.03%:0.2% | Val:Leu:Ala = 98.76%:1.23%:0.01% |
| 26 | ORF8 | 28144 | T:C = 90.47%:9.53% | Leu:Ser = 90.47%:9.53% |
| 27 | N | 28311 | C:T = 98.89%:1.1% | Pro:Leu:Thr:Ser = 98.86%:1.1%:0.02%:0.01% |
| 28 | N | 28580 | G:T = 98.74%:1.26% | Asp:Tyr = 98.74%:1.26% |
| 29 | N | 28851 | G:T:C- = 98.73%:1.26%:0.01% | Ser:Ile:Thr = 98.73%:1.26%:0.01% |
| 30 | N | 28854 | C:T = 98.41%:1.59% | Ser:Leu = 98.41%:1.59% |
| 31 | N | 28863 | C:T = 98.79%:1.21% | Ser:Leu:Thr = 98.78%:1.21%:0.01% |
| 32 | N | 28881 | G:A:T = 74.82%:25.15%:0.02% | Arg:Lys:Ser:Met= 74.78%:25.15%:0.04%:0.02% |
| 33 | N | 28883 | G:C:A:- = 74.89%:25.09%:0.01%:0.01% | Gly:Arg:- = 74.89%:25.1%:0.01% |

# Supplementary Table 3. Non-sense mutation sites in ORF region

| NO | Gene | Location | Base Changes (%) |
| --- | --- | --- | --- |
| 1 | ORF1a | 313 | C:T = 98.84%:1.16% |
| 2 | ORF1a | 1606 | T:G:- = 97.09%:2.89%:0.01% |
| 3 | ORF1a | 1607 | G:- = 97.1%:2.9% |
| 4 | ORF1a | 1608 | A:- = 97.1%:2.9% |
| 5 | ORF1a | 2416 | C:T = 98.16%:1.84% |
| 6 | ORF1a | 3037 | T:C = 70.4%:29.6% |
| 7 | ORF1a | 8782 | C:T = 89.53%:10.47% |
| 8 | ORF1b | 14805 | C:T = 92.13%:7.87% |
| 9 | ORF1b | 15324 | C:T = 97.59%:2.41% |
| 10 | ORF1b | 17247 | T:C:G = 97.16%:2.83%:0.01% |
| 11 | ORF1b | 18060 | C:T:A = 91.9%:8.1%:0.01% |
| 12 | ORF1b | 18877 | C:T = 96.99%:3.01% |
| 13 | ORF1b | 20268 | A:G = 94.76%:5.24% |
| 14 | S | 23731 | C:T = 97.42%:2.58% |
| 15 | S | 24034 | C:T = 98.85%:1.15% |
| 16 | N | 28657 | C:T:A = 98.71%:1.26%:0.03% |
| 17 | N | 28882 | G:A:T = 74.86%:25.09%:0.04%: |

# Supplementary Table 4. ORF region amino acid lost-mutation information list

| No. | Gene | Location | Frequency | No. | Gene | Location | Frequency |
| --- | --- | --- | --- | --- | --- | --- | --- |
| 1 | ORF1a | 362 : 385 | 1/16863 | 41 | S | 21965 : 21973 | 1/16851 |
| 2 | ORF1a | 512 : 517 | 2/16863 | 42 | S | 22259 : 22264 | 1/16851 |
| 3 | ORF1a | 512 : 520 | 11/16863 | 43 | S | 22259 : 22267 | 2/16851 |
| 4 | ORF1a | 512 : 526 | 6/16863 | 44 | S | 23558 : 23575 | 2/16851 |
| 5 | ORF1a | 518 : 523 | 23/16863 | 45 | S | 23570 : 23602 | 1/16851 |
| 6 | ORF1a | 518 : 526 | 16/16863 | 46 | S | 23576 : 23590 | 1/16851 |
| 7 | ORF1a | 686 : 697 | 45/16863 | 47 | ORF3a | 25417 : 25428 | 1/23390 |
| 8 | ORF1a | 1430 : 1435 | 2/16863 | 48 | ORF3a | 25423 : 25434 | 1/23390 |
| 9 | ORF1a | 1580 : 1585 | 1/16863 | 49 | ORF3a | 25429 : 25455 | 1/23390 |
| 10 | ORF1a | 1598 : 1606 | 1/16863 | 50 | ORF3a | 25495 : 25503 | 1/23390 |
| 11 | ORF1a | 1604 : 1609 | 1/16863 | 51 | ORF3a | 25519 : 25536 | 1/23390 |
| 12 | ORF1a | 1604 : 1621 | 1/16863 | 52 | ORF3a | 25621 : 25650 | 1/23390 |
| 13 | ORF1a | 1607 : 1612 | 1/16863 | 53 | ORF3a | 25711 : 25716 | 1/23390 |
| 14 | ORF1a | 3164 : 3172 | 4/16863 | 54 | ORF3a | 25921 : 25950 | 1/23390 |
| 15 | ORF1a | 3248 : 3274 | 1/16863 | 55 | ORF3a | 25993 : 25998 | 1/23390 |
| 16 | ORF1a | 3260 : 3265 | 1/16863 | 56 | ORF3a | 26158 : 26163 | 3/23390 |
| 17 | ORF1a | 3302 : 3322 | 1/16863 | 57 | ORF3a | 26188 : 26205 | 1/23390 |
| 18 | ORF1a | 3305 : 3322 | 3/16863 | 58 | E | 26347 : 26370 | 1/24344 |
| 19 | ORF1a | 3332 : 3343 | 1/16863 | 59 | E | 26359 : 26400 | 1/24344 |
| 20 | ORF1a | 3332 : 3346 | 1/16863 | 60 | E | 26413 : 26418 | 1/24344 |
| 21 | ORF1a | 3863 : 3868 | 3/16863 | 61 | M | 27057 : 27062 | 1/23513 |
| 22 | ORF1a | 3938 : 3946 | 1/16863 | 62 | ORF6 | 27268 : 27294 | 2/24199 |
| 23 | ORF1a | 4880 : 4885 | 2/16863 | 63 | ORF6 | 27274 : 27294 | 1/24199 |
| 24 | ORF1a | 5810 : 5827 | 1/16863 | 64 | ORF6 | 27298 : 27303 | 1/24199 |
| 25 | ORF1a | 6044 : 6049 | 1/16863 | 65 | ORF7a | 27406 : 27489 | 1/21690 |
| 26 | ORF1a | 6374 : 6385 | 1/16863 | 66 | ORF7a | 27586 : 27594 | 1/21690 |
| 27 | ORF1a | 6518 : 6526 | 2/16863 | 67 | ORF7a | 27682 : 27708 | 1/21690 |
| 28 | ORF1a | 6854 : 6862 | 1/16863 | 68 | ORF7a | 27700 : 27705 | 1/21690 |
| 29 | ORF1a | 9722 : 9739 | 1/16863 | 69 | ORF7b | 27768 : 27773 | 3/21953 |
| 30 | ORF1a | 11270 : 11278 | 1/16863 | 70 | ORF7b | 27792 : 27797 | 2/21953 |
| 31 | ORF1a | 11270 : 11281 | 2/16863 | 71 | ORF8 | 28092 : 28097 | 6/24288 |
| 32 | ORF1a | 11282 : 11296 | 1/16863 | 72 | N | 28304 : 28309 | 5/23176 |
| 33 | ORF1a | 12620 : 12625 | 2/16863 | 73 | N | 28880 : 28891 | 1/23176 |
| 34 | ORF1b | 19516 : 19539 | 1/14252 | 74 | N | 28886 : 28903 | 1/23176 |
| 35 | ORF1b | 20410 : 20424 | 1/14252 | 75 | N | 28898 : 28903 | 2/23176 |
| 36 | S | 21737 : 21766 | 2/16851 | 76 | N | 28898 : 28909 | 2/23176 |
| 37 | S | 21929 : 21937 | 1/16851 | 77 | N | 28988 : 28993 | 1/23176 |
| 38 | S | 21953 : 21958 | 1/16851 | 78 | N | 29366 : 29386 | 1/23176 |
| 39 | S | 21953 : 21973 | 1/16851 | 79 | N | 29444 : 29452 | 1/23176 |
| 40 | S | 21959 : 21970 | 3/16851 |  |  |  |  |

# Supplementary Table 5. ORF region amino acid insert-mutation information list

| No. | Gene | Location | Frequency | No. | Gene | Location | Frequency |
| --- | --- | --- | --- | --- | --- | --- | --- |
| 1 | ORF1a | 11083 : 11084 | 33/16863 | 3 | ORF1b | 14607 : 14608 | 1/14252 |
| 2 | ORF1a | 3946 : 3947 | 1/16863 | 4 | ORF1b | 21387 : 21388 | 1/14252 |

# Supplementary Table 6. Prototype sequence ID of haplotypes in Fig.4.

| Haplotype Label | Prototype ID |
| --- | --- |
| L_1 | EPI_ISL_402123 |
| S_1 | EPI_ISL_406801 |
| S_2 | EPI_ISL_408484 |
| S_3 | EPI_ISL_408480 |
| Other_1 | EPI_ISL_408481 |
| S_4 | EPI_ISL_404895 |
| Other_2 | EPI_ISL_408482 |
| Other_3 | EPI_ISL_406036 |
| L_2 | EPI_ISL_406533 |
| L_3 | EPI_ISL_406534 |
| G_1 | EPI_ISL_422425 |
| V_1 | EPI_ISL_415709 |
| L_4 | EPI_ISL_410044 |
| G_2 | EPI_ISL_406862 |
| L_5 | EPI_ISL_421261 |
| Other_4 | EPI_ISL_416331 |
| L_6 | EPI_ISL_410486 |
| S_5 | EPI_ISL_426182 |
| V_2 | EPI_ISL_427810 |
| S_6 | EPI_ISL_413456 |
| GH_1 | EPI_ISL_418218 |
| G_3 | EPI_ISL_420456 |
| S_7 | EPI_ISL_419834 |
| GR_1 | EPI_ISL_412912 |
| S_8 | EPI_ISL_414623 |
| G_4 | EPI_ISL_418251 |
| V_3 | EPI_ISL_413019 |
| GR_2 | EPI_ISL_413997 |
| Other_5 | EPI_ISL_415741 |
| GH_2 | EPI_ISL_418219 |
| V_4 | EPI_ISL_427809 |
| G_5 | EPI_ISL_414019 |
| L_7 | EPI_ISL_414505 |
| GR_3 | EPI_ISL_419559 |
| GH_3 | EPI_ISL_414626 |
| GH_4 | EPI_ISL_418345 |
| GH_5 | EPI_ISL_420791 |
| G_6 | EPI_ISL_413589 |
| GR_4 | EPI_ISL_413647 |
| L_8 | EPI_ISL_413573 |
| GR_5 | EPI_ISL_415702 |
| GR_6 | EPI_ISL_416140 |
| V_5 | EPI_ISL_422865 |
| G_7 | EPI_ISL_414629 |
| L_9 | EPI_ISL_416740 |
| S_9 | EPI_ISL_420786 |
| S_10 | EPI_ISL_428683 |
| Other_6 | EPI_ISL_416032 |
| GR_7 | EPI_ISL_416034 |
| Other_7 | EPI_ISL_417444 |
| Other_8 | EPI_ISL_422407 |
| V_6 | EPI_ISL_426027 |
| S_11 | EPI_ISL_417126 |
| V_7 | EPI_ISL_417268 |
| S_12 | EPI_ISL_418330 |
| S_13 | EPI_ISL_418848 |
| GH_6 | EPI_ISL_420322 |
| Other_9 | EPI_ISL_420339 |
| GR_8 | EPI_ISL_420348 |
| GR_9 | EPI_ISL_420354 |
| G_8 | EPI_ISL_426420 |
| G_9 | EPI_ISL_420321 |
| G_10 | EPI_ISL_415708 |
| GR_10 | EPI_ISL_417992 |
| V_8 | EPI_ISL_415478 |
| G_11 | EPI_ISL_422864 |
| Other_10 | EPI_ISL_436061 |
| G_12 | EPI_ISL_415460 |
| G_13 | EPI_ISL_417969 |
| GR_11 | EPI_ISL_424054 |
| GH_7 | EPI_ISL_424868 |
| Other_11 | EPI_ISL_425845 |
| S_14 | EPI_ISL_428699 |
| GH_8 | EPI_ISL_434455 |
| Other_12 | EPI_ISL_436060 |
| Other_13 | EPI_ISL_436063 |
| GH_9 | EPI_ISL_416521 |
| GH_10 | EPI_ISL_426293 |
| GH_11 | EPI_ISL_430136 |
| G_14 | EPI_ISL_418965 |
| Other_14 | EPI_ISL_430848 |
| L_10 | EPI_ISL_415920 |
| G_15 | EPI_ISL_417727 |
| G_16 | EPI_ISL_417786 |
| G_17 | EPI_ISL_417788 |
| GH_12 | EPI_ISL_421502 |
| S_15 | EPI_ISL_424673 |
| GH_13 | EPI_ISL_427530 |
| GH_14 | EPI_ISL_429161 |
| S_16 | EPI_ISL_436211 |
| S_17 | EPI_ISL_417353 |
| Other_15 | EPI_ISL_419771 |
| GR_12 | EPI_ISL_417509 |
| GR_13 | EPI_ISL_418373 |
| V_9 | EPI_ISL_425648 |
| L_11 | EPI_ISL_425816 |
| V_10 | EPI_ISL_420177 |
| S_18 | EPI_ISL_444532 |
| L_12 | EPI_ISL_418159 |
| S_19 | EPI_ISL_418897 |
| GH_15 | EPI_ISL_419811 |
| G_18 | EPI_ISL_422847 |
| Other_16 | EPI_ISL_422850 |
| GH_16 | EPI_ISL_427603 |
| GR_14 | EPI_ISL_428881 |
| GR_15 | EPI_ISL_417538 |
| G_19 | EPI_ISL_420361 |
| GH_17 | EPI_ISL_421349 |
| GH_18 | EPI_ISL_429869 |
| GH_19 | EPI_ISL_444788 |
| G_20 | EPI_ISL_417534 |
| GH_20 | EPI_ISL_421420 |
| GR_16 | EPI_ISL_421483 |
| V_11 | EPI_ISL_421301 |
| GH_21 | EPI_ISL_426315 |
| S_20 | EPI_ISL_419728 |
| S_21 | EPI_ISL_424199 |
| S_22 | EPI_ISL_426453 |
| GH_22 | EPI_ISL_430229 |
| GH_23 | EPI_ISL_434824 |
| S_23 | EPI_ISL_434827 |
| V_12 | EPI_ISL_419928 |
| Other_17 | EPI_ISL_419965 |
| V_13 | EPI_ISL_425892 |
| G_21 | EPI_ISL_430856 |
| GH_24 | EPI_ISL_429016 |
| GH_25 | EPI_ISL_434871 |
| GR_17 | EPI_ISL_420393 |
| S_24 | EPI_ISL_426942 |
| Other_18 | EPI_ISL_437197 |
| Other_19 | EPI_ISL_420422 |
| GR_18 | EPI_ISL_433350 |
| GH_26 | EPI_ISL_427194 |
| GH_27 | EPI_ISL_434928 |
| GH_28 | EPI_ISL_435450 |
| GH_29 | EPI_ISL_427264 |
| S_25 | EPI_ISL_437495 |
| G_22 | EPI_ISL_425976 |
| GH_30 | EPI_ISL_444683 |
| G_23 | EPI_ISL_422599 |
| Other_20 | EPI_ISL_437199 |
| GH_31 | EPI_ISL_430970 |
| GH_32 | EPI_ISL_443258 |
| GH_33 | EPI_ISL_430974 |
| GR_19 | EPI_ISL_432237 |
| GH_34 | EPI_ISL_435461 |
| GH_35 | EPI_ISL_427451 |
| G_24 | EPI_ISL_433786 |
| GR_20 | EPI_ISL_433844 |
| V_14 | EPI_ISL_437367 |
| GH_36 | EPI_ISL_433448 |
| GR_21 | EPI_ISL_433141 |
| G_25 | EPI_ISL_437746 |
