# Supplementary material for: Deciphering the Subtype Differentiation History of SARS-CoV-2 Based on a New Breadth-First Searching Optimized Alignment Method Over a Global Data Set of 24,768 Sequences
Source: Front Genet. 2021 Jan 11;11:591833. doi: 10.3389/fgene.2020.591833 (PMC7831388; doi:10.3389/fgene.2020.591833)
Supplement: Supplementary file 1 [file Data_Sheet_1.PDF]

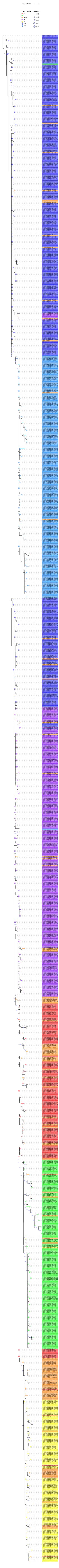
Tree scale: 0.01
Colored ranges
L
S
Other
G
V
GH
GR
bootstrap
0.75
0.79
0.84
0.88
0.92
